# Crystal structure of diethyl 3,3′-[(2-fluoro­phen­yl)methyl­idene]bis­(1*H*-indole-2-carboxyl­ate)

**DOI:** 10.1107/S2056989017015523

**Published:** 2017-10-31

**Authors:** Xin-Hua Lu, Hong-Shun Sun, Yuan Cai, Lu-Lu Chen, Yang-Feng Chen

**Affiliations:** a Targeted MRI Contrast Agents Laboratory of Jiangsu Province, Nanjing Polytechnic Institute, Geguan Road No.265 Nanjing, Nanjing 210048, People’s Republic of China

**Keywords:** crystal structure, bis­indole, MRI, contrast agent

## Abstract

In the title compound, C_29_H_25_FN_2_O_4_, the mean planes of the two indole ring systems (r.m.s. deviations = 0.1392 and 0.0115 Å) are approximately perpendicular to one another, subtending a dihedral angle of 86.0 (5)°; the benzene ring is twisted with respect to the mean planes of the two indole ring systems by 83.3 (2) and 88.1 (4)°, respectively. In the crystal, pairs of N—H⋯O hydrogen bonds link the mol­ecules into centrosymmetric dimers, which are further linked by N—H⋯O hydrogen bonds into supra­molecular chains propagating along the [101] direction.

## Abstract 2

In the title compound, the two indole ring systems are approximately perpendicular to one another, subtending a dihedral angle of 86.0 (5)°. In the crystal, pairs of N—H⋯O hydrogen bonds link the mol­ecules into the inversion dimers, which are further linked by N—H⋯O hydrogen bonds into supra­molecular chains propagated along the *b-*axis direction.

## Chemical context   

Bis(indol­yl)methane derivatives are abundantly present in various terrestrial and marine natural resources (Poter *et al.*, 1977 ▸; Sundberg, 1996 ▸). They are important anti­biotics in the field of pharmaceuticals with diverse activities, such as anti­cancer, anti­leishmanial and anti­hyperlipidemic (Chang *et al.*, 1999 ▸; Ge *et al.*, 1999 ▸). On the other hand, bis­(indoly)methane derivatives can also be used as precursors for MRI necrosis avid contrast agents (Ni, 2008 ▸). In recent years, we have reported the synthesis and crystal structures of some similar bis­(indoly)methane compounds (Sun *et al.*, 2012 ▸, 2015 ▸; Li *et al.*, 2014 ▸; Lu *et al.*, 2014 ▸). Now we report herein another bis­(indoly)methane compound.
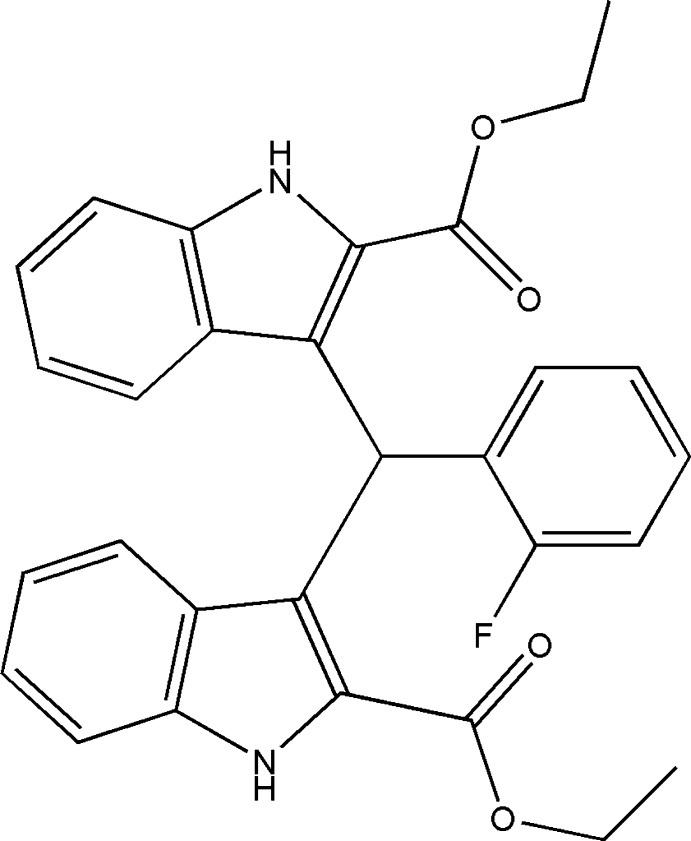


## Structural commentary   

**Figure 1:**
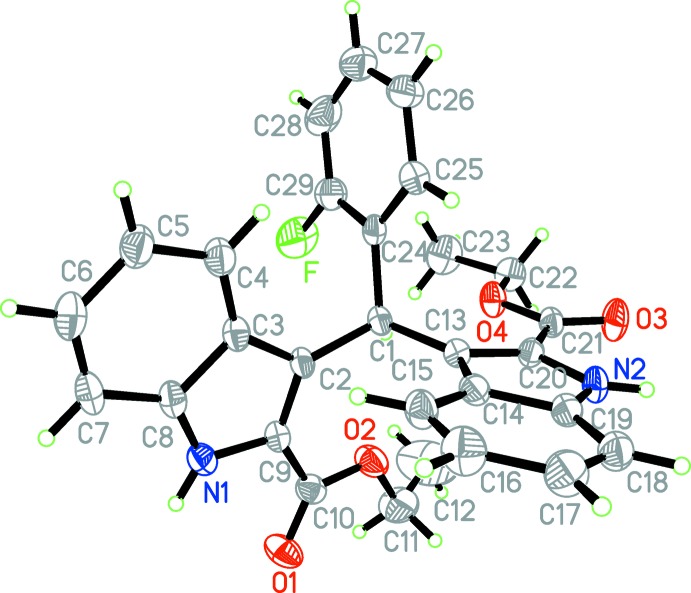
The mol­ecular structure of the title mol­ecule with the atom-labelling scheme. Displacement ellipsoids are drawn at the 30% probability level.

The mol­ecular structure of the title compound is shown in Fig. 1 ▸. The two indole ring systems are nearly perpendicular to each other [dihedral angle = 86.0 (5)°] while the benzene ring (C24–C29) is tilted with respect to the N1/C2–C9 and N2/C13–C20 indole ring systems, making dihedral angles of 83.3 (2) and 88.1 (4)°, respectively. The carboxyl groups are approximately co-planar with the attached indole moieties, the dihedral angles between the carboxyl groups and the mean plane of the attached indole ring system being 9.5 (2) and 7.2 (3)°.

## Supra­molecular features   

**Figure 2:**
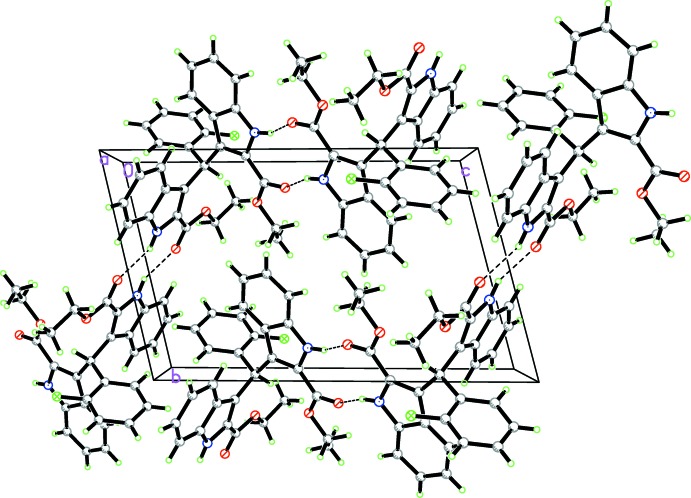
A packing diagram of the title compound. Hydrogen bonds are shown as dashed lines.

In the crystal, pairs of N1—H1*A*⋯O1 hydrogen bonds link the mol­ecules into centrosymmetric dimers, which are further connected by N2—H2*A*⋯O3 hydrogen bonds into supra­molecular zigzag chains propagating along the [101] direction (Table 1 ▸ and Fig. 2 ▸).

## Database survey   

Several similar structures have been reported previously, *i.e*. diethyl 3,3′-(phenyl­methyl­ene)bis­(1*H*-indole-2-carboxyl­ate) (Sun *et al.*, 2012 ▸), dimethyl 3,3′-[(4-fluoro­phen­yl)methyl­ene]bis­(1*H*-indole-2-carboxyl­ate) (Sun *et al.*, 2015 ▸), dimethyl 3,3′-[(4-chloro­phen­yl) methyl­ene]bis­(1*H*-indole-2-carboxyl­ate) (Li *et al.*, 2014 ▸) and 3,3′-[(3-fluoro­phen­yl)methyl­ene]bis­(1*H*-indole-2-carboxyl­ate) (Lu *et al.*, 2014 ▸).

## Synthesis and crystallization   

Ethyl indole-2-carboxyl­ate (1.88 g, 10 mmol) was dissolved in 20 ml ethanol; commercially available 2-fluoro­benzaldehyde (0.62 g, 5 mmol) was added and the mixture was heated to reflux temperature. Concentrated HCl (0.5 ml) was added and the reaction was left for 1 h. After cooling, the white product was filtered off and washed thoroughly with ethanol. The reaction was monitored by TLC (AcOEt:hexane = 1:3). Single crystals of the title compound suitable for X-ray analysis were obtained by slow evaporation of an ethanol solution, yield 90%.

## Refinement   

Crystal data, data collection and structure refinement details are summarized in Table 2 ▸. H atoms were positioned geometrically with N—H = 0.86 Å and C—H = 0.93–0.98 Å, and constrained to ride on their parent atoms with *U*_iso_(H) = *xU*_eq_(C,N), where x = 1.5 for methyl H atoms and 1.2 for all others.

## Supplementary Material

Crystal structure: contains datablock(s) I, global. DOI: 10.1107/S2056989017015523/xu5908sup1.cif

Structure factors: contains datablock(s) I. DOI: 10.1107/S2056989017015523/xu5908Isup2.hkl

Supporting information file. DOI: 10.1107/S2056989017015523/xu5908Isup3.cml

CCDC reference: 1581855

Additional supporting information:  crystallographic information; 3D view; checkCIF report

## Figures and Tables

**Table 1:** Hydrogen-bond geometry (Å, °)

*D*—H⋯*A*	*D*—H	H⋯*A*	*D*⋯*A*	*D*—H⋯*A*
N1—H1*A*⋯O1^i^	0.86	2.10	2.881 (4)	151
N2—H2*A*⋯O3^ii^	0.86	2.07	2.874 (3)	157

Symmetry codes: (i) 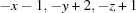; (ii) 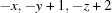.

**Table 2:** Experimental details

Crystal data	
Chemical formula	C_29_H_25_FN_2_O_4_
*M*_r_	484.51
Crystal system, space group	Triclinic, *P* 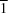
Temperature (K)	293
*a*, *b*, *c* (Å)	8.8000 (18), 9.6610 (19), 15.369 (3)
α, β, γ (°)	75.68 (3), 85.44 (3), 83.68 (3)
*V* (Å^3^)	1256.5 (4)
*Z*	2
Radiation type	Mo *K*α
μ (mm^−1^)	0.09
Crystal size (mm)	0.30 × 0.20 × 0.10

Data collection	
Diffractometer	Nonius CAD-4
Absorption correction	ψ scan (North *et al.*, 1968 ▸)
*T*_min_, *T*_max_	0.973, 0.991
No. of measured, independent and observed [*I* > 2σ(*I*)] reflections	4947, 4621, 2648
*R*_int_	0.037
(sin θ/λ)_max_ (Å^−1^)	0.603

Refinement	
*R*[*F* ^2^ > 2σ(*F* ^2^)], *wR*(*F* ^2^), *S*	0.069, 0.186, 1.00
No. of reflections	4621
No. of parameters	325
No. of restraints	2
H-atom treatment	H-atom parameters constrained
Δρ_max_, Δρ_min_ (e Å^−3^)	0.37, −0.29

Computer programs: *CAD-4 EXPRESS* (Enraf–Nonius, 1994 ▸), *XCAD4* (Harms & Wocadlo, 1995 ▸) and *SHELXTL* (Sheldrick, 2008 ▸).

## supplementary crystallographic information

### Crystal data

**Table:**

C_29_H_25_FN_2_O_4_	*Z* = 2
*M**_r_* = 484.51	*F*(000) = 508
Triclinic, *P*1	*D*_x_ = 1.281 Mg m^−^^3^
Hall symbol: -P 1	Mo *K*α radiation, λ = 0.71073 Å
*a* = 8.8000 (18) Å	Cell parameters from 25 reflections
*b* = 9.6610 (19) Å	θ = 9–12°
*c* = 15.369 (3) Å	µ = 0.09 mm^−^^1^
α = 75.68 (3)°	*T* = 293 K
β = 85.44 (3)°	Block, colorless
γ = 83.68 (3)°	0.30 × 0.20 × 0.10 mm
*V* = 1256.5 (4) Å^3^	

### Data collection

**Table:**

Nonius CAD-4 diffractometer	2648 reflections with *I* > 2σ(*I*)
Radiation source: fine-focus sealed tube	*R*_int_ = 0.037
Graphite monochromator	θ_max_ = 25.4°, θ_min_ = 1.4°
ω/2θ scans	*h* = 0→10
Absorption correction: ψ scan (North *et al.*, 1968)	*k* = −11→11
*T*_min_ = 0.973, *T*_max_ = 0.991	*l* = −18→18
4947 measured reflections	3 standard reflections every 200 reflections
4621 independent reflections	intensity decay: 1%

### Refinement

**Table:**

Refinement on *F*^2^	Primary atom site location: structure-invariant direct methods
Least-squares matrix: full	Secondary atom site location: difference Fourier map
*R*[*F*^2^ > 2σ(*F*^2^)] = 0.069	Hydrogen site location: inferred from neighbouring sites
*wR*(*F*^2^) = 0.186	H-atom parameters constrained
*S* = 1.00	*w* = 1/[σ^2^(*F*_o_^2^) + (0.090*P*)^2^] where *P* = (*F*_o_^2^ + 2*F*_c_^2^)/3
4621 reflections	(Δ/σ)_max_ < 0.001
325 parameters	Δρ_max_ = 0.37 e Å^−^^3^
2 restraints	Δρ_min_ = −0.29 e Å^−^^3^

### Special details

**Table:**

Geometry. All esds (except the esd in the dihedral angle between two l.s. planes) are estimated using the full covariance matrix. The cell esds are taken into account individually in the estimation of esds in distances, angles and torsion angles; correlations between esds in cell parameters are only used when they are defined by crystal symmetry. An approximate (isotropic) treatment of cell esds is used for estimating esds involving l.s. planes.
Refinement. Refinement of F^2^ against ALL reflections. The weighted R-factor wR and goodness of fit S are based on F^2^, conventional R-factors R are based on F, with F set to zero for negative F^2^. The threshold expression of F^2^ > 2sigma(F^2^) is used only for calculating R-factors(gt) etc. and is not relevant to the choice of reflections for refinement. R-factors based on F^2^ are statistically about twice as large as those based on F, and R- factors based on ALL data will be even larger.

### Fractional atomic coordinates and isotropic or equivalent isotropic displacement parameters (Å^2^)

**Table:**

	*x*	*y*	*z*	*U*_iso_*/*U*_eq_	
F	0.1008 (3)	1.1359 (3)	0.64799 (15)	0.0892 (8)	
N1	−0.4074 (3)	1.1149 (3)	0.58021 (16)	0.0475 (7)	
H1A	−0.4582	1.1074	0.5363	0.057*	
O1	−0.3400 (3)	0.8585 (3)	0.53292 (16)	0.0649 (7)	
C1	−0.1205 (3)	0.9767 (3)	0.75362 (18)	0.0355 (7)	
H1B	−0.0473	0.9276	0.7172	0.043*	
N2	−0.1865 (3)	0.6560 (3)	0.93763 (16)	0.0444 (7)	
H2A	−0.1563	0.5744	0.9718	0.053*	
O2	−0.1486 (3)	0.7970 (3)	0.62507 (17)	0.0638 (7)	
C2	−0.2422 (3)	1.0600 (3)	0.69080 (18)	0.0380 (7)	
O3	0.1216 (3)	0.5759 (2)	0.90831 (16)	0.0634 (7)	
C3	−0.3183 (4)	1.1998 (3)	0.68783 (19)	0.0417 (8)	
O4	0.1394 (2)	0.7613 (2)	0.79087 (14)	0.0507 (6)	
C4	−0.3137 (4)	1.3049 (3)	0.7359 (2)	0.0529 (9)	
H4A	−0.2488	1.2893	0.7830	0.064*	
C5	−0.4062 (4)	1.4314 (4)	0.7129 (2)	0.0606 (10)	
H5A	−0.4018	1.5015	0.7443	0.073*	
C6	−0.5063 (4)	1.4570 (4)	0.6435 (2)	0.0605 (10)	
H6A	−0.5680	1.5433	0.6299	0.073*	
C7	−0.5152 (4)	1.3575 (4)	0.5952 (2)	0.0551 (9)	
H7A	−0.5813	1.3751	0.5486	0.066*	
C8	−0.4223 (4)	1.2284 (4)	0.6177 (2)	0.0467 (8)	
C9	−0.2988 (3)	1.0132 (3)	0.62295 (19)	0.0393 (7)	
C10	−0.2664 (4)	0.8840 (4)	0.5891 (2)	0.0467 (8)	
C11	−0.1055 (5)	0.6707 (4)	0.5897 (3)	0.0861 (14)	
H11A	−0.1634	0.5930	0.6234	0.103*	
H11B	−0.1327	0.6921	0.5275	0.103*	
C12	0.0510 (7)	0.6258 (8)	0.5943 (5)	0.178 (3)	
H12A	0.0736	0.5438	0.5691	0.267*	
H12B	0.0778	0.6005	0.6559	0.267*	
H12C	0.1089	0.7021	0.5609	0.267*	
C13	−0.1830 (3)	0.8604 (3)	0.82871 (18)	0.0353 (7)	
C14	−0.3323 (3)	0.8555 (3)	0.8754 (2)	0.0405 (8)	
C15	−0.4693 (4)	0.9485 (4)	0.8693 (2)	0.0479 (8)	
H15A	−0.4763	1.0351	0.8261	0.057*	
C16	−0.5897 (4)	0.9099 (4)	0.9272 (2)	0.0622 (10)	
H16A	−0.6795	0.9711	0.9231	0.075*	
C17	−0.5836 (4)	0.7812 (4)	0.9929 (2)	0.0620 (10)	
H17A	−0.6685	0.7585	1.0318	0.074*	
C18	−0.4548 (4)	0.6886 (4)	1.0008 (2)	0.0528 (9)	
H18A	−0.4508	0.6023	1.0442	0.063*	
C19	−0.3295 (4)	0.7259 (3)	0.9426 (2)	0.0422 (8)	
C20	−0.0989 (3)	0.7352 (3)	0.87031 (19)	0.0376 (7)	
C21	0.0629 (4)	0.6816 (3)	0.8594 (2)	0.0418 (8)	
C22	0.2998 (4)	0.7133 (4)	0.7765 (2)	0.0571 (10)	
H22A	0.3098	0.6184	0.7649	0.069*	
H22B	0.3553	0.7094	0.8293	0.069*	
C23	0.3614 (5)	0.8187 (5)	0.6976 (3)	0.0827 (13)	
H23A	0.4676	0.7904	0.6858	0.124*	
H23B	0.3513	0.9120	0.7100	0.124*	
H23C	0.3053	0.8218	0.6460	0.124*	
C24	−0.0307 (3)	1.0737 (3)	0.7890 (2)	0.0382 (7)	
C25	−0.0470 (4)	1.0918 (3)	0.8758 (2)	0.0465 (8)	
H25A	−0.1161	1.0400	0.9167	0.056*	
C26	0.0363 (5)	1.1848 (4)	0.9036 (3)	0.0654 (11)	
H26A	0.0206	1.1967	0.9620	0.078*	
C27	0.1401 (5)	1.2582 (4)	0.8465 (3)	0.0695 (11)	
H27A	0.1957	1.3209	0.8654	0.083*	
C28	0.1636 (5)	1.2406 (4)	0.7610 (3)	0.0780 (12)	
H28A	0.2370	1.2889	0.7216	0.094*	
C29	0.0772 (4)	1.1507 (4)	0.7339 (2)	0.0564 (9)	

### Atomic displacement parameters (Å^2^)

**Table:**

	*U*^11^	*U*^22^	*U*^33^	*U*^12^	*U*^13^	*U*^23^
F	0.111 (2)	0.0973 (18)	0.0577 (14)	−0.0417 (15)	0.0308 (13)	−0.0126 (13)
N1	0.0534 (18)	0.0491 (17)	0.0401 (15)	−0.0023 (14)	−0.0122 (13)	−0.0089 (13)
O1	0.0653 (16)	0.0822 (19)	0.0573 (15)	0.0048 (14)	−0.0190 (13)	−0.0360 (14)
C1	0.0391 (17)	0.0353 (16)	0.0296 (15)	0.0022 (14)	−0.0054 (13)	−0.0046 (13)
N2	0.0459 (16)	0.0382 (15)	0.0412 (15)	0.0039 (13)	−0.0051 (13)	0.0028 (12)
O2	0.0672 (17)	0.0560 (15)	0.0732 (17)	0.0115 (13)	−0.0284 (14)	−0.0253 (13)
C2	0.0433 (18)	0.0373 (17)	0.0297 (16)	−0.0008 (14)	−0.0064 (14)	−0.0011 (13)
O3	0.0520 (15)	0.0496 (14)	0.0694 (16)	0.0128 (12)	−0.0009 (13)	0.0132 (12)
C3	0.0471 (19)	0.0410 (18)	0.0325 (16)	0.0018 (15)	−0.0026 (14)	−0.0030 (14)
O4	0.0452 (14)	0.0510 (14)	0.0462 (13)	0.0065 (11)	0.0017 (11)	0.0003 (11)
C4	0.063 (2)	0.047 (2)	0.0464 (19)	0.0075 (18)	−0.0100 (17)	−0.0090 (16)
C5	0.075 (3)	0.044 (2)	0.060 (2)	0.0071 (19)	−0.009 (2)	−0.0092 (17)
C6	0.062 (3)	0.049 (2)	0.060 (2)	0.0139 (18)	−0.001 (2)	−0.0031 (19)
C7	0.049 (2)	0.057 (2)	0.048 (2)	0.0079 (18)	−0.0100 (17)	0.0050 (18)
C8	0.047 (2)	0.047 (2)	0.0386 (18)	0.0018 (16)	−0.0059 (15)	0.0017 (15)
C9	0.0410 (18)	0.0382 (17)	0.0355 (17)	0.0015 (14)	−0.0061 (14)	−0.0039 (14)
C10	0.047 (2)	0.055 (2)	0.0384 (18)	−0.0085 (17)	−0.0013 (16)	−0.0107 (16)
C11	0.098 (4)	0.056 (3)	0.116 (4)	0.008 (2)	−0.022 (3)	−0.044 (3)
C12	0.137 (6)	0.164 (7)	0.272 (10)	0.044 (5)	−0.060 (6)	−0.135 (7)
C13	0.0357 (17)	0.0361 (17)	0.0333 (16)	−0.0020 (13)	−0.0032 (13)	−0.0072 (13)
C14	0.0410 (19)	0.0422 (18)	0.0381 (17)	−0.0016 (15)	−0.0077 (15)	−0.0082 (14)
C15	0.0401 (19)	0.047 (2)	0.051 (2)	0.0038 (16)	−0.0063 (16)	−0.0036 (16)
C16	0.043 (2)	0.068 (3)	0.070 (3)	0.0091 (19)	0.0035 (19)	−0.014 (2)
C17	0.050 (2)	0.075 (3)	0.057 (2)	−0.009 (2)	0.0058 (18)	−0.010 (2)
C18	0.048 (2)	0.053 (2)	0.053 (2)	−0.0056 (18)	−0.0059 (17)	−0.0028 (17)
C19	0.0422 (19)	0.0435 (19)	0.0398 (18)	−0.0041 (15)	−0.0044 (15)	−0.0071 (15)
C20	0.0380 (18)	0.0335 (17)	0.0381 (17)	−0.0039 (14)	−0.0022 (14)	−0.0024 (14)
C21	0.046 (2)	0.0375 (18)	0.0383 (18)	0.0026 (15)	−0.0074 (15)	−0.0039 (15)
C22	0.044 (2)	0.060 (2)	0.063 (2)	0.0032 (18)	0.0058 (18)	−0.0133 (19)
C23	0.065 (3)	0.091 (3)	0.084 (3)	−0.011 (2)	0.020 (2)	−0.013 (3)
C24	0.0384 (18)	0.0332 (16)	0.0377 (17)	0.0026 (14)	−0.0060 (14)	0.0000 (13)
C25	0.048 (2)	0.046 (2)	0.0441 (19)	−0.0018 (16)	−0.0078 (16)	−0.0071 (15)
C26	0.077 (3)	0.055 (2)	0.069 (3)	0.000 (2)	−0.025 (2)	−0.022 (2)
C27	0.066 (3)	0.062 (3)	0.082 (3)	−0.014 (2)	−0.016 (2)	−0.014 (2)
C28	0.066 (3)	0.064 (3)	0.097 (3)	−0.023 (2)	0.001 (3)	0.000 (2)
C29	0.062 (2)	0.057 (2)	0.049 (2)	−0.0120 (19)	−0.0008 (18)	−0.0093 (18)

### Geometric parameters (Å, º)

**Table:**

F—C29	1.360 (4)	C12—H12A	0.9600
N1—C8	1.349 (4)	C12—H12B	0.9600
N1—C9	1.372 (4)	C12—H12C	0.9600
N1—H1A	0.8600	C13—C20	1.384 (4)
O1—C10	1.205 (4)	C13—C14	1.446 (4)
C1—C24	1.511 (4)	C14—C19	1.412 (4)
C1—C13	1.513 (4)	C14—C15	1.417 (4)
C1—C2	1.522 (4)	C15—C16	1.351 (4)
C1—H1B	0.9800	C15—H15A	0.9300
N2—C20	1.365 (4)	C16—C17	1.393 (5)
N2—C19	1.367 (4)	C16—H16A	0.9300
N2—H2A	0.8600	C17—C18	1.358 (5)
O2—C10	1.326 (4)	C17—H17A	0.9300
O2—C11	1.456 (4)	C18—C19	1.384 (4)
C2—C9	1.380 (4)	C18—H18A	0.9300
C2—C3	1.432 (4)	C20—C21	1.472 (4)
O3—C21	1.200 (3)	C22—C23	1.486 (5)
C3—C4	1.402 (4)	C22—H22A	0.9700
C3—C8	1.424 (4)	C22—H22B	0.9700
O4—C21	1.325 (4)	C23—H23A	0.9600
O4—C22	1.453 (4)	C23—H23B	0.9600
C4—C5	1.376 (4)	C23—H23C	0.9600
C4—H4A	0.9300	C24—C29	1.374 (4)
C5—C6	1.395 (5)	C24—C25	1.382 (4)
C5—H5A	0.9300	C25—C26	1.383 (5)
C6—C7	1.364 (5)	C25—H25A	0.9300
C6—H6A	0.9300	C26—C27	1.347 (5)
C7—C8	1.396 (4)	C26—H26A	0.9300
C7—H7A	0.9300	C27—C28	1.363 (6)
C9—C10	1.460 (4)	C27—H27A	0.9300
C11—C12	1.400 (6)	C28—C29	1.372 (5)
C11—H11A	0.9700	C28—H28A	0.9300
C11—H11B	0.9700		
			
C8—N1—C9	109.5 (3)	C14—C13—C1	129.9 (3)
C8—N1—H1A	125.2	C19—C14—C15	117.5 (3)
C9—N1—H1A	125.2	C19—C14—C13	107.3 (3)
C24—C1—C13	111.8 (2)	C15—C14—C13	135.1 (3)
C24—C1—C2	112.5 (2)	C16—C15—C14	119.2 (3)
C13—C1—C2	113.1 (2)	C16—C15—H15A	120.4
C24—C1—H1B	106.3	C14—C15—H15A	120.4
C13—C1—H1B	106.3	C15—C16—C17	122.1 (3)
C2—C1—H1B	106.3	C15—C16—H16A	119.0
C20—N2—C19	109.8 (2)	C17—C16—H16A	119.0
C20—N2—H2A	125.1	C18—C17—C16	120.7 (3)
C19—N2—H2A	125.1	C18—C17—H17A	119.7
C10—O2—C11	116.5 (3)	C16—C17—H17A	119.7
C9—C2—C3	106.3 (3)	C17—C18—C19	118.4 (3)
C9—C2—C1	125.3 (3)	C17—C18—H18A	120.8
C3—C2—C1	128.4 (3)	C19—C18—H18A	120.8
C4—C3—C8	117.8 (3)	N2—C19—C18	130.4 (3)
C4—C3—C2	135.7 (3)	N2—C19—C14	107.4 (3)
C8—C3—C2	106.4 (3)	C18—C19—C14	122.2 (3)
C21—O4—C22	116.5 (2)	N2—C20—C13	110.1 (3)
C5—C4—C3	119.3 (3)	N2—C20—C21	117.4 (3)
C5—C4—H4A	120.3	C13—C20—C21	132.3 (3)
C3—C4—H4A	120.3	O3—C21—O4	122.5 (3)
C4—C5—C6	121.6 (4)	O3—C21—C20	123.4 (3)
C4—C5—H5A	119.2	O4—C21—C20	114.0 (3)
C6—C5—H5A	119.2	O4—C22—C23	107.0 (3)
C7—C6—C5	121.0 (3)	O4—C22—H22A	110.3
C7—C6—H6A	119.5	C23—C22—H22A	110.3
C5—C6—H6A	119.5	O4—C22—H22B	110.3
C6—C7—C8	118.2 (3)	C23—C22—H22B	110.3
C6—C7—H7A	120.9	H22A—C22—H22B	108.6
C8—C7—H7A	120.9	C22—C23—H23A	109.5
N1—C8—C7	129.9 (3)	C22—C23—H23B	109.5
N1—C8—C3	108.1 (3)	H23A—C23—H23B	109.5
C7—C8—C3	122.0 (3)	C22—C23—H23C	109.5
N1—C9—C2	109.7 (3)	H23A—C23—H23C	109.5
N1—C9—C10	116.5 (3)	H23B—C23—H23C	109.5
C2—C9—C10	133.9 (3)	C29—C24—C25	115.3 (3)
O1—C10—O2	122.7 (3)	C29—C24—C1	120.1 (3)
O1—C10—C9	123.0 (3)	C25—C24—C1	124.6 (3)
O2—C10—C9	114.3 (3)	C24—C25—C26	121.9 (3)
C12—C11—O2	112.8 (4)	C24—C25—H25A	119.1
C12—C11—H11A	109.0	C26—C25—H25A	119.1
O2—C11—H11A	109.0	C27—C26—C25	120.3 (4)
C12—C11—H11B	109.0	C27—C26—H26A	119.8
O2—C11—H11B	109.0	C25—C26—H26A	119.8
H11A—C11—H11B	107.8	C26—C27—C28	120.0 (4)
C11—C12—H12A	109.5	C26—C27—H27A	120.0
C11—C12—H12B	109.5	C28—C27—H27A	120.0
H12A—C12—H12B	109.5	C27—C28—C29	118.9 (4)
C11—C12—H12C	109.5	C27—C28—H28A	120.5
H12A—C12—H12C	109.5	C29—C28—H28A	120.5
H12B—C12—H12C	109.5	F—C29—C28	118.1 (4)
C20—C13—C14	105.4 (2)	F—C29—C24	118.3 (3)
C20—C13—C1	124.6 (3)	C28—C29—C24	123.6 (4)
			
C24—C1—C2—C9	153.2 (3)	C19—C14—C15—C16	0.0 (5)
C13—C1—C2—C9	−79.0 (4)	C13—C14—C15—C16	−178.2 (3)
C24—C1—C2—C3	−25.5 (4)	C14—C15—C16—C17	0.0 (6)
C13—C1—C2—C3	102.3 (3)	C15—C16—C17—C18	−0.3 (6)
C9—C2—C3—C4	−179.3 (4)	C16—C17—C18—C19	0.6 (5)
C1—C2—C3—C4	−0.4 (6)	C20—N2—C19—C18	−178.6 (3)
C9—C2—C3—C8	1.5 (3)	C20—N2—C19—C14	−0.1 (3)
C1—C2—C3—C8	−179.6 (3)	C17—C18—C19—N2	177.7 (3)
C8—C3—C4—C5	−1.3 (5)	C17—C18—C19—C14	−0.6 (5)
C2—C3—C4—C5	179.5 (3)	C15—C14—C19—N2	−178.3 (3)
C3—C4—C5—C6	0.9 (5)	C13—C14—C19—N2	0.3 (3)
C4—C5—C6—C7	−0.6 (6)	C15—C14—C19—C18	0.3 (5)
C5—C6—C7—C8	0.6 (5)	C13—C14—C19—C18	178.9 (3)
C9—N1—C8—C7	178.2 (3)	C19—N2—C20—C13	−0.1 (4)
C9—N1—C8—C3	0.2 (4)	C19—N2—C20—C21	175.6 (3)
C6—C7—C8—N1	−178.8 (3)	C14—C13—C20—N2	0.3 (3)
C6—C7—C8—C3	−1.1 (5)	C1—C13—C20—N2	177.5 (3)
C4—C3—C8—N1	179.6 (3)	C14—C13—C20—C21	−174.5 (3)
C2—C3—C8—N1	−1.1 (4)	C1—C13—C20—C21	2.7 (5)
C4—C3—C8—C7	1.4 (5)	C22—O4—C21—O3	0.8 (5)
C2—C3—C8—C7	−179.2 (3)	C22—O4—C21—C20	−179.8 (3)
C8—N1—C9—C2	0.7 (4)	N2—C20—C21—O3	−2.8 (5)
C8—N1—C9—C10	−178.8 (3)	C13—C20—C21—O3	171.7 (3)
C3—C2—C9—N1	−1.4 (3)	N2—C20—C21—O4	177.8 (3)
C1—C2—C9—N1	179.7 (3)	C13—C20—C21—O4	−7.8 (5)
C3—C2—C9—C10	178.0 (3)	C21—O4—C22—C23	−179.5 (3)
C1—C2—C9—C10	−0.9 (5)	C13—C1—C24—C29	157.1 (3)
C11—O2—C10—O1	3.4 (5)	C2—C1—C24—C29	−74.4 (4)
C11—O2—C10—C9	−176.4 (3)	C13—C1—C24—C25	−22.3 (4)
N1—C9—C10—O1	−7.4 (5)	C2—C1—C24—C25	106.2 (3)
C2—C9—C10—O1	173.2 (3)	C29—C24—C25—C26	2.0 (5)
N1—C9—C10—O2	172.4 (3)	C1—C24—C25—C26	−178.5 (3)
C2—C9—C10—O2	−7.0 (5)	C24—C25—C26—C27	−1.8 (5)
C10—O2—C11—C12	150.0 (5)	C25—C26—C27—C28	−0.2 (6)
C24—C1—C13—C20	−80.3 (4)	C26—C27—C28—C29	1.8 (6)
C2—C1—C13—C20	151.4 (3)	C27—C28—C29—F	179.1 (3)
C24—C1—C13—C14	96.1 (3)	C27—C28—C29—C24	−1.5 (6)
C2—C1—C13—C14	−32.1 (4)	C25—C24—C29—F	179.0 (3)
C20—C13—C14—C19	−0.4 (3)	C1—C24—C29—F	−0.5 (5)
C1—C13—C14—C19	−177.3 (3)	C25—C24—C29—C28	−0.4 (5)
C20—C13—C14—C15	177.9 (3)	C1—C24—C29—C28	−179.8 (3)
C1—C13—C14—C15	1.0 (6)		

### Hydrogen-bond geometry (Å, º)

**Table:**

*D*—H···*A*	*D*—H	H···*A*	*D*···*A*	*D*—H···*A*
N1—H1*A*···O1^i^	0.86	2.10	2.881 (4)	151
N2—H2*A*···O3^ii^	0.86	2.07	2.874 (3)	157

Symmetry codes: (i) −*x*−1, −*y*+2, −*z*+1; (ii) −*x*, −*y*+1, −*z*+2.
